# The Sumo coach problem

**DOI:** 10.1007/s10058-022-00316-4

**Published:** 2022-11-04

**Authors:** Daniel Rehsmann

**Affiliations:** University of Vienna, 1090 Vienna, Austria

**Keywords:** Seeding, Contests, Teams, Latin square games, C7, D7, H4, M3

## Abstract

We address the optimal allocation of stochastically dependent resource bundles to a set of simultaneous contests. For this purpose, we study a modification of the Colonel Blotto Game called the Tennis Coach Problem. We devise a thoroughly probabilistic method of payoff representation and fully characterize equilibria in this class of games. We further formalize the idea of strategic team training in a comparative static setting. The problem applies to several distinct economic interactions but seems most prevalent in team sports with individual matches, for instance, in Tennis and Sumo.

## Introduction

This paper analyzes the optimal allocation of stochastically dependent resource bundles to a collection of interconnected contests. Two players competitively allocate their bundles across a finite number of contests. A single contest is won in expectation by the player who submits a stochastically dominant random variable; the probabilistic distance increases in the realized resource differential. Each player’s expected payoff for the whole game is a combination of the probabilities of winning single contests. The optimal allocation of the available resources involves strategic considerations. Should a player try to narrowly win as many contests as possible, or should she “tank” some of them? Intuition suggests that an optimal allocation strategy must take each resource bundle’s strength into account and involve some randomness. We answer how to optimally allocate random variables among the set of such interconnected contests.

Our main contribution is to characterize the set of Nash equilibria in all non-trivial parameter settings of the defined class of games and to prove the existence of mixed-strategy equilibria in tournaments involving three or more competitors. Although all equilibria share common characteristics, they vary in terms of pure strategy support. The characterization is closely linked to a combinatorial concept, namely to the notion of “Latin squares.”^1^ Following Ferguson (2020), we define our game as a “Latin square game” and provide necessary and sufficient conditions for equilibria in all finite two-person zero-sum Latin square games. We then use generic properties of the equilibria in our game to answer questions of optimal marginal alterations of a player’s bundles in a comparative static analysis, postulating equilibrium play.

The studied interaction shares some aspects with the Colonel Blotto game, introduced by Borel (1921), in which two players compete across several battlefields by partitioning a fixed number of troops among them. While a Colonel Blotto game allows for all (or all discrete) partitions of the total resources to be allocated, the interaction studied in this paper restricts the players to assign a priori fixed but random partitions to component contests. We formalize a version of Snyder’s (1989) definition of a simultaneous multi-component contest, altering the allocational restrictions accordingly.

In a more applied context, Hamilton and Romano (1998) and Arad (2012) study similar problems referred to as the “Tennis Coach Problem.” We approach a similar problem but assume stochastic resource bundles that render the payoff representation entirely probabilistic. The modeled interaction seems applicable to several economic settings where the allocation of stochastic resources to a set of contested situations is the primary strategic element. Examples include organizational competitions and races between firms or electoral campaigning across several districts. The defined interaction also seems prevalent in team sports with individual matches such as Tennis, Sumo, Chess, and many others. In a broader context, our analysis can be applied to settings similar to the Colonel Blotto problem but tends to reflect less continuous assignment assumptions in situations where individuals are matched to distinct tasks.

In our narrative, the described strategic elements are embedded in the context of a Sumo tournament, where team coaches assign their competitors to a set of distinct bilateral matches. A rank of a Sumo competitor reflects past performance in tournaments (see, e.g., Duggan and Levitt 2002); the probability of winning a specific match, however, depends on various factors (see, e.g., Bleekley et al. 2010). Addressing the complexity of Sumo tournaments, we thus assume that coaches are uninformed about actual strength realizations of the competitors as the assignment is chosen. We model the situation as a one-shot zero-sum game; in particular, the coaches of two teams—each of the latter endowed with idiosyncratic skill distributions—simultaneously announce their team members’ assignments to independent matches. The competitors feature commonly known ranks, and in expectation, a stronger competitor wins a match. All winning probabilities of the independent matches are then combined into an overall probability of winning the team competition, which the team coaches maximize by choosing an optimal allocation strategy.

We structure the remainder of the paper as follows and start by relating our contribution to the existing literature. We introduce the model in Sects. 2 and characterize the equilibria in Sect. 3. We apply a comparative static analysis in Sect. 4 and discuss possible extensions and limitations of our model in Sect. 5. All proofs can be found in the “Appendix”.

### Related literature

This paper mainly contributes to the literature on multi-battle contests. Such games share the characteristic that contenders meet each other on multiple battlefields, with discrete battles on each front. General overviews of multi-battle contests are provided by, e.g., Vojnović (2016) and Fu and Wu (2019). The classical literature on simultaneous multi-battle contests follows the original formulation of the Colonel Blotto game, due to Borel (1921), which features prominently in the early literature. Early contributions include, e.g., Gross and Wagner (1950), Blackett (1958) and Tukey (1949). More recent work that contributes to “Blotto-type” games examines asymmetries in resources or objectives (e.g., Roberson 2006; Kvasov 2007; Hart 2008; Avrahami and Kareev 2009; Kovenock and Roberson 2020) or alternative definitions of success (e.g., Golman and Page 2009; Kovenock and Roberson 2010, 2020).

Since the present model differs in its strategic setting markedly from a Colonel Blotto game, it can be embedded more accurately in the literature on multi-battle contests between teams.Such contests are defined as “team contests with multiple pairwise battles” by Fu et al. (2015). A sequential formalization of such multi-battle contests dates back to Harris and Vickers (1987), who analyze the combination of component contests in the context of a dynamic R &D race. A simultaneous multi-battle contest was introduced by Snyder (1989), where two parties compete in parallel elections by allocating campaign resources to a set of legislative districts. In contrast to this formalization, our model restricts the allocation of resources to fixed partitions and follows the intuition of Hamilton and Romano (1998). Their basic setting as a two-player, zero-sum game, where team coaches assign their (ranked) competitors to a set of discrete tournaments, is also maintained in our contribution. This paper differs from their contribution by introducing a more general probabilistic method of payoff formalization linked to the concept of Latin squares. Furthermore, we assume that at the time of assignment, coaches are uninformed about the actual strength of each competitor. Moreover, we provide a more comprehensive characterization of equilibria in such games.

Arad (2012) explores a setting similar to Hamilton and Romano (1998) in a primarily experimental study, in which coaches assign four heterogeneously skilled players to four positions. The game-setting is formalized as an *n*-player constant sum game, with non-probabilistic payoffs on the distinct playing slots. Equilibria and experimental behavior are analyzed from the perspective of a boundedly rational, mainly behavioral approach. A setting more closely linked to the Colonel Blotto literature is analyzed by Rinott et al. (2012), studying a sequential setup. Players allocate a finite amount of resources to individual team members, engaging in a sequence of one-on-one fights. The particular team members’ participation in such a tournament is analyzed by Fu et al. (2015). Their paper contrasts from our work since they assume that the players’ assignments to battlefields are fixed, while we exclude the team members’ effort choices from our analysis while assigning players to battlefields strategically.

Adopting Ferguson (2020)’s definition of Latin square games as distinct versions of constant-sum games, some results of this paper also contribute to that literature. A necessary and sufficient condition for the existence of Nash equilibria in this model can be generalized to all two-player Latin square games. To the best of our knowledge, Ferguson (2020) is the only existing discussion of Latin square games in the literature.

Finally, our comparative static analysis contributes to research on improving team performance. Strategic considerations in the training of teams remain mainly unformalized in the management, organizational, and theoretical literature. Team performance and leadership issues are studied in the context of contests by Gershkov and Schweinzer (2021). Both the psychology literature (e.g., Salas and Cannon-Bowers 2002) and research in human resource management (e.g., Campbell and Kuncel 2002) frequently contribute to the analysis of team performance. An example of a meta-study in the management literature is Salas et al. (2008).

## The model

We consider a tournament in which two teams $J\in \{A,B\}$, each composed of $n \ge 2$ competitors indexed $i\in N=\{1,2,\ldots ,n\}$, compete in *n* single matches. The tournament is treated as a one-shot zero-sum game, in which each team coach assigns every team member of her team (in a potentially stochastic way) to match indices. Two competitors generate payoff expectations based on probability masses $p^J_{k} \in [0,1]$ to win a distinct match $k \in N$. The probability that a team wins a specific match between two competitors is a function of the two individual competitors stochastic strengths facing each other in a given match. The distinct matches are combined to a team’s overall probability of winning the whole tournament, $\rho ^J$, as a function of probabilities to win on individual matches. Team coaches maximize their team’s overall expected probability of winning the matches. Each team’s combined skill is represented by an independent and commonly known continuous probability distribution $F^J$. Let $f^J=F^{\prime J}$ represent the associated probability density function with finite support $[0,\bar{\theta }]$, $\bar{\theta }\in \mathbb {R}_+$ and $f^J>0$ everywhere. We assume a certain extent of randomness in an individual competitor’s realized strength. Although unsure about a competitor’s strength realization in a specific match, the team coaches can order them stochastically. We assume that the individual competitor’s strengths are independent draws from a team’s skill probability distribution. A sorting in increasing order is represented by the dependent random variables $\Theta ^J_{(i)}$, featuring the density of the *i*th lowest among *n* order statistics:

$$\begin{aligned} f_{\Theta ^J_{(i)}}(\theta )=n{n-1 \atopwithdelims ()i-1} f^J(\theta )F^J(\theta )^{i-1} (1-F^J(\theta ))^{n-i}. \end{aligned}$$

This implies that team members can be ranked according to their stochastic strength, with a higher index corresponding to higher random competitor strength.^2^ The resulting set of stochastic player strengths available to a coach *J* is $\{\Theta ^J_{(1)},\Theta ^J_{(2)},\ldots ,\Theta ^J_{(n)}\}$, in which $\Theta ^J_{(1)}<_{\text {s.t.}}\Theta ^J_{(2)}<_{\text {s.t.}}\ldots <_{\text {s.t.}}\Theta ^J_{(n)}$ in the usual stochastic order $<_{\text {s.t.}}$, i.e.,^3^

$$\begin{aligned} \mathbb {P}[\Theta ^J_{(k)}\le \theta ]>\mathbb {P}[\Theta ^J_{(l)}\le \theta ],\quad \forall \theta \in [0, \bar{\theta }]\hbox { and any }1\le k <l \le n. \end{aligned}$$

Hence, for $k<l$, $\Theta ^J_{(k)}$ is less likely than $\Theta ^J_{(l)}$ to take on higher strength values, where “higher” means any value greater than $\theta$.

A team coach chooses a distinct player slot $k \in N$ for every member *i* of their team *J*. Such a bijective assignment of *n* players to *n* slots is called seeding, and all possible seedings define the strategy set of a team coach. Since both coaches can choose every possible permutation of slots, the set of pure strategies contains *n*! elements and is identical for both coaches. A pure strategy is denoted by a permutation $s_l \in S^J$ with $l\in N!=\{1,\ldots ,n!\}$ of the numbered competitors over *n* playing slots. Let $a_x\in S^A$ represent a pure strategy of team coach *A* and $b_y\in S^B$ a pure strategy of team coach *B*, respectively. We order the permutations (for notational convenience) lexicographically; thus $s_1$ denotes the first permutation of player indices in lexicographic order. The Cartesian product $S^A \times S^B$ defines the set of all possible “lineups” $L=\{(a_1,b_1),(a_1,b_2),\ldots ,(a_{n!},b_{n!})\}$ with cardinality $n!^2$. Let $\Theta _{(i)}(a_x(k))$ denote the stochastic strength of the player *i* seeded at slot *k* by coach *A* in her strategy $a_x$. Likewise, $\Theta _{(j)}(a_x(k))$ denotes the stochastic strength of the $k^{\text {th}}$-seeded player *j* by coach *B* in her strategy $b_y$. A specific lineup is denoted by $(a_x,b_y)$, defined to be a *n*-dimensional nested tuple

1 $$\begin{aligned} (a_x,b_y)= & {} \langle [\Theta (a_x(1)),\Theta (b_y(1))],\ldots , [\Theta (a_x(k)),\Theta (b_y(k))],\ldots ,\nonumber \\&[\Theta (a_x(n)),\Theta (b_y(n))]\rangle , \end{aligned}$$

in which the pair $[\Theta (a_x(k)),\Theta (b_y(k))]$ represents the stochastic strengths of the two players seeded *k*th by the strategy pair $(a_x,b_y)$ in their match for slot *k*.

The individual matches among the competitors are treated as contests based on strength realizations of the competitors assigned to a distinct match.^4^ For that purpose, denote by $\theta ^A,\theta ^B$ the corresponding realizations of the *k*th seeded players. We define team *J*’s probability of winning a single match indexed *k* as a function

$$\begin{aligned} q^J (\theta ^A,\theta ^B), \end{aligned}$$

on which we impose the following assumptions, covering conventional functional forms of contest success functions (CSF) (see, e.g., Fu and Wu 2019):

1. exclusivity: the probability to win a distinct slot is verifiable with quality-dependent probabilities summing to $q^A(\theta ^A,\theta ^B)+q^B(\theta ^A,\theta ^B)=1$;
2. symmetry: the probability $q^J(\theta ^A,\theta ^B)$ is equal to $\,\!^{1}\!/_{2}$ for identical qualities $\theta ^A=\theta ^B$;
3. responsiveness: the probability $q^A(\theta ^A,\theta ^{B})$ is increasing in $\theta ^A$ and decreasing in $\theta ^{A}$ with $q^J(\theta ^A,\theta ^B)<1$ for $\theta ^B>0$.

Since a coach is uninformed about the strength realizations of the competitors, a team coach expects to win match *k* with probability

$$\begin{aligned} p^J (\Theta (a_x(k)),\Theta (b_y(k)))=\mathbb {E}[q^J (\Theta (a_x(k)),\Theta (b_y(k)))]. \end{aligned}$$

We assume separability among across battles and define the probability that a team wins the whole tournament, given a distinct lineup $l(a_x,b_y)$ as the average of winning on separate playing slots

$$\begin{aligned} \frac{1}{n}\sum _{k=1}^{n}q^J (\theta (a_x(k)),\theta (b_y(k))). \end{aligned}$$

Hence, coaches value all matches equally and only care about the sum of all winning probabilities of individual matches $p^A(\cdot )$, normalized by the number of matches *n*. By linearity of the expectation, a coach expects

2 $$\begin{aligned} \rho ^J(a_x,b_y)= & {} \mathbb {E}\left[ \frac{1}{n}\sum _{k=1}^{n}q^J (\Theta (a_x(k)),\Theta (b_y(k)))\right] \nonumber \\ {}= & {} \frac{1}{n}\sum _{k=1}^{n}p^J (\Theta (a_x(k)),\Theta (b_y(k))). \end{aligned}$$

Observe that by (P1), we have $q^A(\theta ^A,\theta ^B)=1-q^B(\theta ^A,\theta ^B)$, which (again by the linearity of the expectation) implies $p^A(\Theta (a_x(k)),\Theta (b_y(k)))=1-p^B(\Theta (a_x(k)),\Theta (b_y(k)))$, and thus also $\rho ^A(a_x,b_y)=1-\rho ^B(a_x,b_y)$, defining a constant-sum game.^5^ Accordingly, we drop the superscripts on the payoffs to simplify notation.

Denote by $\Phi = (\rho (a_x,b_y))_{x\in N!,y\in N!}$ the game’s strategic-form matrix as a result of the available *n*! strategies to each coach. For the case of pure strategies, only one lineup occurs with strictly positive probability, and the maximization problem for the coach of team *A* can be defined as a best-response: given a pure strategy $b_y\in S^B$, coach *A* chooses a pure strategy $a_x\in S^A$ to achieve a most favorable lineup

$$\begin{aligned} \underset{a_x\in S^A}{arg\,max\,}\ \rho (a_x,b_y). \end{aligned}$$

We introduce mixed strategies as points in the unit simplex $\alpha \in \Delta (S^A)$, $\beta \in \Delta (S^B)$. The probability with which a team plays the seeding $s_l$ in a mixed strategy is denoted by $\alpha _x$, $\beta _y$, as the $l^{th}$ dimension of the associated unit simplex, providing the expected mixed strategy payoff defined as

3 $$\begin{aligned} \pi (\alpha ,\beta )=\sum _{x=1}^{n!}\sum _{y=1}^{n!}\alpha _x\rho (a_x,b_y)\beta _y. \end{aligned}$$

We modify the overall probability that coach *A* wins the whole tournament by allowing for mixtures accordingly and define the two coaches’ optimization problems as

$$\begin{aligned} \underset{\alpha \in \Delta (S^A)}{arg\,max\,} \pi (\alpha ,\beta )\text { for coach { A}, }\quad \underset{\beta \in \Delta (S^B)}{arg\,min\,} \pi (\alpha ,\beta )\text { for coach { B}.} \end{aligned}$$

Before finishing this section, we summarize the timing interaction, which unfolds as follows: 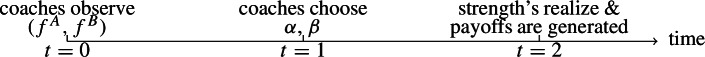


### Example of a three competitor tournament

#### Example 1

We now introduce a specific example of a three-competitor tournament to illustrate the mechanics of our model. (In later examples, we will continue to build on this first example setting.) The expected strengths of the participating competitors are independent draws from the continuous probability distributions $F^J$, $J\in \{A,B\}$. In the present example, team *A*’s skill, $F^A$, is represented by the symmetric triangular distribution with support $x \in [0,1]$, denoted by $\mathcal {T}[0,1]$. Team *B*’s skill, $F^B$, is represented by the uniform distribution with equivalent support, denoted by $\mathcal {U}[0,1]$. The expected player strengths take the (order-statistic-defined) values:

$$\begin{aligned}&\mathbb {E}[\Theta _{(1)}^A]=0.325, \mathbb {E}[\Theta _{(2)}^A]=0.5,\mathbb {E}[\Theta _{(3)}^A]=0.675,\\&\mathbb {E}[\Theta _{(1)}^B]=0.250, \mathbb {E}[\Theta _{(2)}^B]=0.5,\mathbb {E}[\Theta _{(3)}^B]=0.750. \end{aligned}$$

Team coaches assign team members to playing slots $k\in \{1,2,3\}$. Table 1 illustrates the strategy space $S^J$ available to each coach; as indicated, there are six such pure strategies per coach. A pair of two pure strategies constitute a lineup $(a_x,b_y)$. For example, $(a_1,b_2)$ constitutes the lineup

**Table 1:** Pure strategy set $S^J$ for a team of $n=3$ players using the notation $\Theta _{(\text {player})}$

	Playing slots		
	$k=1$	$k=2$	$k=3$
*Strategies*			
$s_1$	$\Theta _{(1)}$	$\Theta _{(2)}$	$\Theta _{(3)}$
$s_2$	$\Theta _{(1)}$	$\Theta _{(3)}$	$\Theta _{(2)}$
$s_3$	$\Theta _{(2)}$	$\Theta _{(1)}$	$\Theta _{(3)}$
$s_4$	$\Theta _{(2)}$	$\Theta _{(3)}$	$\Theta _{(1)}$
$s_5$	$\Theta _{(3)}$	$\Theta _{(1)}$	$\Theta _{(2)}$
$s_6$	$\Theta _{(3)}$	$\Theta _{(2)}$	$\Theta _{(1)}$

$$\begin{aligned} (a_1,b_2)= & {} \langle [\Theta (a_1(1)),\Theta (b_2(1)))],[\Theta (a_1(2)),\Theta (b_2(2))],[\Theta (a_1(3)),\Theta (b_2(3))]\rangle \\ {}= & {} \langle [\Theta _{(1)}^A,\Theta _{(1)}^B],[\Theta _{(2)}^A,\Theta _{(3)}^B],[\Theta _{(3)}^A,\Theta _{(2)}^B]\rangle . \end{aligned}$$

We embed the lottery CSF as the probability of winning distinct matches. Hence, on slot 1, player *A* wins with probability

$$\begin{aligned} p(\Theta (a_1(1),\Theta (b_2(1))=\mathbb {E}\left[ \frac{\Theta _{(1)}^A}{\Theta _{(1)}^A+\Theta _{(1)}^B}\right] \approx 0.598. \end{aligned}$$

Given the lineup $(a_1,b_2)$, player *A* wins the tournament with probability

$$\begin{aligned}&\rho (a_1,b_2)\\&\quad =\frac{1}{3}\left( \mathbb {E}\left[ \frac{\Theta _{(1)}^A}{\Theta _{(1)}^A+\Theta _{(1)}^B}\right] +\mathbb {E}\left[ \frac{\Theta _{(2)}^A}{\Theta _{(2)}^A+\Theta _{(3)}^B}\right] +\mathbb {E}\left[ \frac{\Theta _{(3)}^A}{\Theta _{(3)}^A+\Theta _{(2)}^B}\right] \right) \approx 0.531. \end{aligned}$$

Given team *B*’s pure strategy, for instance $b_2$, team *A*’s pure strategy best responses yield:

$$\begin{aligned}&\rho (a_2,b_2)\approx 0.531 {>} \rho (a_1,b_2)\approx 0.530 {>} \rho (a_3,b_2)\approx 0.529 {>}\\&\rho (a_4,b_2)\approx 0.527 {>} \rho (a_5,b_2)\approx 0.524 {>} \rho (a_6,b_2)\approx 0.521. \end{aligned}$$

Team *A* chooses the pure strategy $a_x$ which solves the maximization problem

$$\begin{aligned} a_2= \underset{a_x\in S^A}{arg\,max\,}\ \rho (a_x,b_2). \end{aligned}$$

If team *B* plays, e.g., the mixed strategy $\bar{\beta }=\langle 1/2,0,0,0,0,1/2\rangle$, team *A* maximizes

$$\begin{aligned} \pi (\alpha ,\bar{\beta })=\frac{1}{2}\sum _{x=1}^{n!}\alpha _x\left( \rho (a_x,b_1)+\rho (a_x,b_6)\right) , \end{aligned}$$

by choosing some mixed strategy $\alpha$. Observe that pure strategy responses of team *A* to mixed strategy $\bar{\beta }$ yield payoffs

$$\begin{aligned}&\rho (a_2,\bar{\beta }) \approx 0.530 \approx \rho (a_4,\bar{\beta }){>} \\&\rho (a_1,\bar{\beta }) \approx 0.528 \approx \rho (a_6,\bar{\beta }){>} \\&\rho (a_3,\bar{\beta }) \approx 0.524 \approx \rho (a_5,\bar{\beta }). \end{aligned}$$

It is easy to see that any convex combination of the form

$$\begin{aligned} \underset{\alpha \in \Delta (S^A)}{arg\,max\,}\ \pi (\alpha ,\beta )= \langle 0,\lambda ,0,(1-\lambda ),0,0\rangle \hbox { with } \lambda \in [0,1], \end{aligned}$$

solves team *A*’s maximization problem, i.e., by splitting the entire probability mass between the strategies $a_2=[\Theta _{(1)},\Theta _{(3)},\Theta _{(2)}]$ and $a_4=[\Theta _{(2)},\Theta _{(3)},\Theta _{(1)}]$.

## Equilibrium characterization

This section characterizes the set of equilibrium strategies in tournaments of arbitrary size. We describe the reasons for the occurrence of multiple mixed-strategy equilibria and trace typical properties of equilibrium strategies. The defined contest involves some symmetry in the payoffs, which induces the game to share features of a particular type of zero-sum game. Following Hamilton and Romano (1998) and Ferguson (2020), we characterize such tournaments as two-player Latin square games. Combined with the combinatorial structure of the strategy spaces, such games feature distinct properties, which we utilize to identify equilibrium strategies.

### Preliminary results

Before moving on to specific properties of the payoff matrix, we begin this section by formally transferring properties of the embedded CSF onto the expected probability of winning a particular match. This ensures that the studied interaction is indeed a constant sum game. Moreover, the described monotonicity of the expectation is central for our comparative analysis.

#### Lemma 1

For any measurable function $q(\theta ^A,\theta ^B)$ satisfying (Q1–Q3), $\mathbb {E}[q^J (\Theta _{(i)}^B,\Theta _{(j)}^B)]$ satisfies following properties

1. exclusivity: the espexted probability to win a distinct slot is verifiable with quality-dependent probabilities summing to $\mathbb {E}[q^A (\Theta _{(i)}^A,\Theta _{(j)}^B)]+\mathbb {E}[q^B (\Theta _{(i)}^A,\Theta _{(j)}^B)]=1$;
2. symmetry: the expected probability $\mathbb {E}[q^A (\Theta _{(i)}^A,\Theta _{(j)}^B)]$ is equal to $\,\!^{1}\!/_{2}$ for identical random variables $\Theta ^A_{(i)}=\Theta ^B_{(j)}$;
3. responsiveness: the expected probability $\mathbb {E}[q^A (\Theta _{(i)}^A,\Theta _{(j)}^B)]$ is increasing in *i* and decreasing in *j*, for $i,j\in \{1,\ldots ,n\}$.

The characterization of equilibrium properties in the remainder of the paper refers to a specific structure of the payoff matrix $\Phi$. To avoid confusion in our definitions, we stick to the definition introduced by Hamilton and Romano (1998), named “pure strategy equivalence,” which is equivalent to the definition of a “Latins square game,” introduced by Ferguson (2020):

#### Definition 1

(PSE) Each row and column of the payoff matrix $\Phi$ contains the same expected payoffs.

Some equilibrium properties rely on a stronger concept, which refers to the full combinatorial definition of a Latin square. Following Colbourn and Dinitz (1996), a Latin square is defined as an $n\times n$ array *L*, in which each cell contains a single element from a set of cardinality *n*, such that each element occurs exactly once in each row and exactly once in each column.^6^ We characterize games with an accordingly structured payoff matrix as games satisfying “Latin square properties” (LSP):

#### Definition 2

(LSP) Each row and column of the payoff matrix $\Phi$ contains the same expected payoffs. Furthermore, each expected payoff occurs exactly once in each row and exactly once in each column.

In this subsection, we want to establish that tournaments of arbitrary team size, as defined above, feature (PSE). In addition, we show that certain contest success functions may imply that such tournaments also satisfy (LSP). We start with observing a distinct characteristic of the studied game: two pairs of strategies (i.e., lineups), $(a_x,b_y)$ and $(a_{x'},b_{y'})$, are payoff-equivalent if $(a_x,b_y)$ and $(a_{x'},b_{y'})$ only differ in the slots at which the same players meet each other. This is the case if the matches (i.e., the pairs $(a_{x'}(k),b_{y'}(k))$) in $(a_{x'},b_{y'})$ are a permutation of $(a_x,b_y)$ over the *k* playing slots.

#### Lemma 2

Two lineups, $(a_x,b_y)$ and $(a_{x'},b_{y'})$, are equivalent in payoffs if $(a_{x'},b_{y'})$ is a permutation of the matches in $(a_{x},b_{y})$ over the *k* playing slots. The pure-strategy payoff function $\rho (a_x,b_y)$ is, therefore, a non-injective mapping.

Thus, a team’s payoff function is independent of the slot at which individual matches are played. We collect such payoff-equivalent lineups in pairwise disjoint subsets. Let $\mathscr {L}=\{\mathscr {L}_1,\ldots ,\mathscr {L}_{n!}\}$ be a partition of the set of lineups *L*, such that a subset $\mathscr {L}_i \subset L$, $i\in N!$ is defined as

4 $$\begin{aligned} \mathscr {L}_i:=\{(a_x,b_y)|(a_x,b_y)\text { is a permutation of }(a_x,b_i)\text { over }k\}. \end{aligned}$$

Every subset $\mathscr {L}_i$ consequently contains *n*! lineups $(a_x,b_y)$ that result in equivalent payoffs, hence making the coaches payoff-indifferent among lineups $(a_x,b_y)$ and $(a_{x'},b_{y'})$ that are elements of the same subset $\mathscr {L}_i$, as stated in Lemma 2

$$\begin{aligned} \rho ((a_x,b_y)\in \mathscr {L}_i)=p((a_{x'},b_{y'}){\in } \mathscr {L}_i) \hbox {for }x\ne x'\hbox { and }y\ne y'. \end{aligned}$$

We demonstrate the payoff equivalence of lineups in a certain lineup class in the following example.

#### Example 2

Table 2 shows the possible pairings in the $n=3$ competitor tournament arising from Example 1. There are 36 possible pairings, which are elements of 6 payoff distinct subsets $\{\mathscr {L}_1,\ldots ,\mathscr {L}_6\}$. For instance, the lineup $(a_1,b_2)\in \mathscr {L}_2$ is payoff equivalent to $(a_2,b_1)\in \mathscr {L}_2$:

$$\begin{aligned} \rho (a_1,b_2)= & {} \frac{1}{3}\left( p(\Theta _{(1)}^A,\Theta _{(1)}^B)+p(\Theta _{(2)}^A,\Theta _{(3)}^B)+p(\Theta _{(3)}^A,\Theta _{(2)}^B)\right) \\ {}= & {} \frac{1}{3}\left( p(\Theta _{(1)}^A,\Theta _{(1)}^B)+p(\Theta _{(3)}^A,\Theta _{(2)}^B)+p(\Theta _{(2)}^A,\Theta _{(3)}^B)\right) = \rho (a_2,b_1), \end{aligned}$$

but may yield a different payoffs than $(a_1,b_1)\in \mathscr {L}_1$:

$$\begin{aligned} \rho (a_1,b_2)= & {} \frac{1}{3}\left( p(\Theta _{(1)}^A,\Theta _{(1)}^B)+p(\Theta _{(2)}^A,\Theta _{(3)}^B)+p(\Theta _{(3)}^A,\Theta _{(2)}^B)\right) \\\ne & {} \frac{1}{3}\left( p(\Theta _{(1)}^A,\Theta _{(1)}^B)+p(\Theta _{(2)}^A,\Theta _{(2)}^B)+p(\Theta _{(3)}^A,\Theta _{(3)}^B)\right) = \rho (a_1,b_1). \end{aligned}$$

Hence, lineups are payoff equivalent if the same players merely meet at different slots.

**Table 2:** Lineups in a game with $n=3$ players

Team B						
	$b_1$	$b_2$	$b_3$	$b_4$	$b_5$	$b_6$
*Team A*						
$a_1$	$\mathscr {L}_1$	$\mathscr {L}_2$	$\mathscr {L}_3$	$\mathscr {L}_4$	$\mathscr {L}_5$	$\mathscr {L}_6$
$a_2$	$\mathscr {L}_{2}$	$\mathscr {L}_{1}$	$\mathscr {L}_{4}$	$\mathscr {L}_{3}$	$\mathscr {L}_{6}$	$\mathscr {L}_{5}$
$a_3$	$\mathscr {L}_{3}$	$\mathscr {L}_{5}$	$\mathscr {L}_{1}$	$\mathscr {L}_{6}$	$\mathscr {L}_{2}$	$\mathscr {L}_{4}$
$a_4$	$\mathscr {L}_{5}$	$\mathscr {L}_{3}$	$\mathscr {L}_{6}$	$\mathscr {L}_{1}$	$\mathscr {L}_{4}$	$\mathscr {L}_{2}$
$a_5$	$\mathscr {L}_{4}$	$\mathscr {L}_{6}$	$\mathscr {L}_{2}$	$\mathscr {L}_{5}$	$\mathscr {L}_{1}$	$\mathscr {L}_{3}$
$a_6$	$\mathscr {L}_{6}$	$\mathscr {L}_{4}$	$\mathscr {L}_{5}$	$\mathscr {L}_{2}$	$\mathscr {L}_{3}$	$\mathscr {L}_{1}$

Two lineups that are elements of different subsets $\mathscr {L}_i$ can confront the team coaches with different expected payoffs. If we have payoff-equivalence of two arbitrary lineups that are elements of distinct lineup classes, i.e., there exists some

$$\begin{aligned} \rho ((a_x,b_y)\in \mathscr {L}_i)=\rho ((a_{x'},b_{y'}){\in } \mathscr {L}_j),\quad \hbox { for }i\ne j, \end{aligned}$$

the tournament satisfies (PSE), but violates (LSP). If, as a result of the embedded CSF, the lineup classes result in unique payoffs, i.e., we have

$$\begin{aligned} \rho ((a_x,b_y)\in \mathscr {L}_i){\ne } \rho ((a_{x'},b_{y'})\in \mathscr {L}_j),\quad \forall i\ne j, \end{aligned}$$

the resulting payoff matrix additionally satisfies (LSP). We provide a proof for this statement in the following lemma.

#### Lemma 3

If $\mathscr {P}=\{\rho ((a_x,b_y)\in \mathscr {L}_i):i\in N!\}$ is a weakly ordered set, the payoff matrix $\Phi$ satisfies (PSE). If $\mathscr {P}$ is a strictly ordered set, the payoff matrix $\Phi$ satisfies (LSP).

We illustrate the concepts of (PSE) and (LSP) by embedding two different CSF, i.e., functional representations, to win single contests. As shown in the following example, these concepts generally depend on the monotonicity properties of the embedded CSF. We illustrate that (PSE) arises by embedding a weakly monotonic CSF, i.e., assuming that $q(\theta ^A,\theta ^B)$ is weakly increasing in $\theta ^A$ and weakly decreasing in $\theta ^B$, into our previous examples. In contrast, integrating a strictly monotonic CSF — in general—results in a payoff-matrix satisfying (LSP).

#### Example 3

Consider again the tournament outlined in Example 1 Embedding the all-pay auction CSF as the probability of winning a distinct match *k*, defined as:

$$\begin{aligned} q(\theta ^A,\theta ^B)={\left\{ \begin{array}{ll} 1 &{} \theta ^A>\theta ^B\\ \,\!^{1}\!/_{2} &{} \theta ^A=\theta ^B\\ 0 &{} \theta ^A<\theta ^B \end{array}\right. }, \end{aligned}$$

yields, e.g., for strategy pairs, $(a_1,b_1)\in \mathscr {L}_1$ and $(a_1,b_6)\in \mathscr {L}_6$, the expected payoff

$$\begin{aligned} \rho (a_1,b_1)\approx \,\!^{1}\!/_{3}(0.65+0.5+0.35)=\frac{1}{2}=\,\!^{1}\!/_{3}(0.05+0.5+0.95)\approx \rho (a_1,b_6). \end{aligned}$$

The lineup $(a_1,b_2)\in \mathscr {L}_2$, however, results in a different expected payoff

$$\begin{aligned} \rho (a_1,b_2)\approx \,\!^{1}\!/_{3}(0.65+0.15+0.73)\approx 0.512. \end{aligned}$$

The lineup classes result in payoffs

$$\begin{aligned}&\{\rho (\mathscr {L}_1)=.500,\rho (\mathscr {L}_2)=.512,\rho (\mathscr {L}_3)=.488,\rho (\mathscr {L}_4)=.456,\rho (\mathscr {L}_5)=.543,\rho (\mathscr {L}_6)=.500\}.\\&\rho ((a_x,b_y)\in \mathscr {L}_1)=.500,\rho ((a_x,b_y)\in \mathscr {L}_2)=.512,\rho ((a_x,b_y)\in \mathscr {L}_3)=.488,\\&\rho ((a_x,b_y)\in \mathscr {L}_4)=.456,\rho ((a_x,b_y)\in \mathscr {L}_5)=.543,\rho ((a_x,b_y)\in \mathscr {L}_6)=.500. \end{aligned}$$

Entering these values into Table 2, demonstrates, that the payoff matrix $\Phi$ indeed satisfies (PSE) since every row and column contains the same elements. Furthermore, observe that the same payoffs may arise in distinct lineup classes (as for $\rho ((a_x,b_y)\in \mathscr {L}_1)=\rho ((a_x,b_y)\in \mathscr {L}_6)$), thus contradicting (LSP).

Embedding the lottery CSF as the probability to win a distinct match *k*, defined as

$$\begin{aligned} q(\theta ^A,\theta ^B)=\frac{\theta ^A}{\theta ^A+\theta ^B}, \end{aligned}$$

results in payoffs

$$\begin{aligned}&\rho ((a_x,b_y)\in \mathscr {L}_1)=.531,\rho ((a_x,b_y)\in \mathscr {L}_2)=.530,\rho ((a_x,b_y)\in \mathscr {L}_3)=.527, \\&\rho ((a_x,b_y)\in \mathscr {L}_4)=.521,\rho ((a_x,b_y)\in \mathscr {L}_5)=.529,\rho ((a_x,b_y)\in \mathscr {L}_6)=.524. \end{aligned}$$

Since payoffs are different in every lineup class $\mathscr {L}_i$, the matrix $\Phi$ in addition satisfies (LSP), since every payoff occurs exactly once in each row and once in each column.

Although strict responsiveness of the embedded contest success function may guarantee (LSP) for “typical” parameter settings of such games, certain skill distributions induce payoff equivalence of two strategies. In a $n=2$ competitor tournament such payoff-equivalence can occur, e.g., if $\rho (a_1,b_1)=\rho (a_1,b_2)$. In that case, we have pure strategy payoffs

$$\begin{aligned} \frac{1}{2}\left( p(\Theta _{(1)}^A,\Theta _{(1)}^B)+p(\Theta _{(2)}^A,\Theta _{(2)}^B)\right) =\frac{1}{2}\left( p(\Theta _{(1)}^A,\Theta _{(2)}^B)+p(\Theta _{(2)}^A,\Theta _{(1)}^B)\right) . \end{aligned}$$

The occurrence of such pairwise equalities in tournaments of arbitrary size depends on the specific form of the embedded CSF. For instance, embedding the lottery CSF in a two-competitor tournament, the above equality holds if and only if $\mathbb {E}[\Theta _{(1)}^A\Theta _{(2)}^A]=\mathbb {E}[\Theta _{(1)}^B\Theta _{(2)}^B]$.

### Nash equilibria

As a consequence of (PSE), each pure strategy of a coach confronts the opponent with the same set of feasible payoffs. Thus, if team *B* decides to play any pure strategy $b_y$, team *A* could always find a pure strategy to obtain its most preferred lineup. Due to the zero-sum property of the game, the presence of such a pure strategy best response leads to the conclusion that pure strategy equilibria are only possible in a very small subclass of parameter settings. As Hamilton and Romano (1998) show, pure strategy equilibria indeed only exist under very stringent conditions, more precisely, if the payoffs are equal for every possible lineup, i.e.,

$$\begin{aligned} \rho (a_1,b_1)=\rho (a_1,b_2)=\ldots =\rho (a_{n!},b_{n!}). \end{aligned}$$

As this case seems uninteresting, we exclude such trivial parameter settings from consideration in our further characterization of equilibrium strategies. The same reasoning also implies that mixed strategies which result in unequal realization probabilities of player assignments cannot be part of a Nash equilibrium. As a starting point, we use a result established by Hamilton and Romano (1998) and Ferguson (2020), which confirms that equal probability mixing over the entire strategy space always constitutes an equilibrium of a zero-sum game satisfying (PSE). Due to the zero-sum properties, min-max solutions correspond to the Nash equilibria. Hence, we begin the characterization of equilibria by outlining the relatively simple structure of the value of such games.

#### Lemma 4

In a two player zero-sum game satisfying (PSE) of dimension $n!\times n!$ and pure strategy payoff $\rho (a_x,b_y)$, the value *V* of the game is

$$\begin{aligned} \frac{1}{n!}\sum _{x=1}^{n!}\rho (a_x,b_y)=V=\frac{1}{n!}\sum _{y=1}^{n!}\rho (a_x,b_y). \end{aligned}$$

Observing the structure of the payoff matrix in games satisfying (PSE) allows to refine the indifference condition of equilibrium profiles in the following way:

#### Proposition 1

A profile $\beta ^{*}$ constitutes an optimal strategy of an arbitrary finite two-person zero-sum game with (PSE) of size $n!\times n!$, if and only if the opposing team *A* is indifferent among all their pure strategies, i.e.,

5 $$\begin{aligned} \pi (a_x,\beta ^{*})=\sum _{y=1}^{n!}\beta _y \rho (a_x,b_y)=\sum _{y=1}^{n!}\beta _y \rho (a_{x'},b_y)=\pi (a_{x'},\beta ^{*}) \forall a_x\ne a_{x'} \in S^A. \end{aligned}$$

The observation that a distinct equilibrium strategy profile in games with pure strategy equivalence makes the opponent indifferent among all pure strategies provides the necessary structure to identify other mixed strategy equilibria. In many other games with pure strategy equivalence, bilateral uniform mixing over the entire pure strategy space may constitute the only way to establish such a form of indifference. However, the combinatorial structure of the pure strategy spaces invokes some redundancies in the payoffs that allow for the presence of multiple equilibria. We demonstrate this observation in the following example.

#### Example 4

Take again the payoffs calculated using a lottery CSF in Example 3. The payoffs in the lineup classes are

$$\begin{aligned} \mathscr {L}_1\approx 0.531,\mathscr {L}_2\approx 0.530,\mathscr {L}_3\approx 0.527,\mathscr {L}_4\approx 0.521,\mathscr {L}_5\approx 0.529,\mathscr {L}_6\approx 0.524. \end{aligned}$$

A mixed strategy profile of team *B* that makes the row player indifferent among all of their pure strategies is, e.g., the profile

$$\begin{aligned} \beta _2=\langle 1/3,0,0,1/3,1/3,0\rangle . \end{aligned}$$

Team *A*’s pure strategy responses all yield expected payoff of

$$\begin{aligned} \pi (a_x,\beta _2)\approx 0.527. \end{aligned}$$

A decomposition of the pure strategy reactions of team *A* illustrates the logic. Team *A*’s response, e.g., $a_1$ yields the decomposed payoff

$$\begin{aligned} \pi (a_1,\beta _2)=\frac{1}{3}\rho ((a_1,b_1){\in } \mathscr {L}_1)+\frac{1}{3}\rho ((a_1,b_4)\in \mathscr {L}_4)+\frac{1}{3}\rho ((a_1,b_5)\in \mathscr {L}_5). \end{aligned}$$

Team *A* can achieve its most favorable lineup $(a_1,b_5)\in \mathscr {L}_5$ with a probability of 1/3. The reaction $a_1$ is punished because team *A*’s least favorable lineup $(a_1,b_4)\in \mathscr {L}_4$ is also played with a probability of 1/3. The reaction $a_2$ to $\beta _2$ yields the same expected payoff and can be decomposed into

$$\begin{aligned} \pi (a_2,\beta _2)=\frac{1}{3}\rho ((a_2,b_1){\in } \mathscr {L}_2)+\frac{1}{3}\rho ((a_2,b_4)\in \mathscr {L}_3)+\frac{1}{3}\rho ((a_2,b_5)\in \mathscr {L}_6). \end{aligned}$$

Both teams achieve their second to most favorable lineups with a probability of 1/3, while team *B* can only achieve its third to most favorable lineup $(a_2,b_5)\in \mathscr {L}_6$. All pure strategy responses $a_x$ to $\beta _2$ yield the same expected payoff since all players of team *B* are assigned in expectation with the same probability to every slot *k*. Observe that this is also the case if player *B* plays every pure strategy with probability 1/*n*!. Hence, team *A* is indifferent also among all its pure strategies when facing $\beta _2$.

As illustrated in the previous example, we may see the occurrence of multiple mixed strategy equilibria due to the combinatorial redundancies in the teams’ pure strategy spaces. However, if we decompose the payoff generation into specific playing slots, we can identify some shared properties of equilibrium strategies. Recall that the probability of winning the tournament given two pure strategies is defined as the average of winning probabilities on distinct playing slots. Thus, plugging in (2) into (3) gives for a pure strategy $a_x$ the payoff

$$\begin{aligned} \pi (a_x,\beta ) =\sum _{y=1}^{n!}\beta _y \frac{1}{n}\sum _{k=1}^{n} p(\Theta (a_x(k)),\Theta (b_y(k))). \end{aligned}$$

In the next proposition, we want to establish that if player *B* plays optimal according to Proposition 1, player *A* is indifferent between the slots, some competitor $\Theta _{(i)}$ is assigned to. We denote team *A*’s payoff obtained on a distinct playing slot *k*, given its strategy $a_x$ and team *B*’s mixed strategy $\beta$, by the $k^{th}$ vertical sum of $\pi (a_x,\beta )$, denoting $\Theta _{(i)}=\Theta (a_x(k))$

$$\begin{aligned} \pi (a_x,\beta ,k)= & {} \frac{1}{n}\sum _{y=1}^{n!}\beta _y p(\Theta (a_x(k)),\Theta (b_y(k)))\\ {}= & {} \frac{1}{n}\sum _{y=1}^{n!}\beta _yp(\Theta _{(i)},\Theta (b_y(k)))=\pi (\Theta _{(i)},\beta ,k). \end{aligned}$$

#### Proposition 2

A sufficient condition for a strategy profile $\beta$ to satisfy Proposition 1 is

6 $$\begin{aligned} \pi (\Theta _{(i)},\beta ,k) = \pi (\Theta _{(i)},\beta ,k') \forall i,k\ne k'\in N. \end{aligned}$$

In games satisfying (LSP) this condition is also necessary for Proposition 1.

A direct consequence of this observation is that an equilibrium profile thus has to result in a somehow balanced assignment, i.e., seeding each competitor with the same probability to every playing slot.

#### Proposition 3

A strategy profile satisfies Proposition 2 if every competitor $\Theta _{(i)}$ is assigned with equal probability to every playing slot *k*. If the game matrix satisfies (LSP), this condition is also necessary.

As illustrated in Example 4, uniform randomization over specific pure strategies, while others are played with probability 0, can also satisfy Proposition 1 and thus constitute an equilibrium game strategy. We define a mixed strategy profile that satisfies Proposition 1 with support of cardinality *n* as a “minimal support equilibrium” strategy.

#### Proposition 4

Let $\hat{S}^B \subset S^B$ denote a proper subset of *n* strategies $\hat{b}_y$, such that there exists exactly one $(\dot{\exists })$ strategy that assigns competitor $\Theta ^B_{(i)}$ to playing slot *k*. Let $\hat{\beta }_y$ denote the mixed strategy dimension of $\beta$ associated to a pure strategy $\hat{b}_y$. Uniform randomization over a such subset $\hat{S}^B$, i.e. $\hat{\beta }_y=1/n$, satisfies Proposition 2, and thus constitutes a minimal support equilibrium strategy of the game.

The cardinality of possible subsets, which can constitute a minimal support equilibrium strategy, refers to an open topic in combinatorics. By definition, a set $\hat{S}^{B}$ contains precisely *n* strategies, such that every player is assigned precisely once to a particular playing slot *k*. A subset $\hat{S}^B$ thus defines a Latin square, where (due to the lexicographic order of strategies) the first row occurs in a natural order. Colbourn and Dinitz (1996) define such combinatorial structures as “half-normalized Latin squares”. In tournaments of size *n*, there are thus as many minimal support equilibrium strategies as possible half-normalized Latin squares. For tournaments with up to $n=11$ competitors, we provide the set of possible minimal support equilibrium in Table 3.

**Table 3:** Possible minimal support equilibrium strategies in games up to $n=11$ competitors, based on McKay and Wanless (2005)

*n*	Minimal support equilibrium strategies
1	1
2	1
3	2
4	24
5	1, 344
6	1, 128, 960
7	12, 198, 297, 600
8	2, 697, 818, 265, 354, 240
9	15, 224, 734, 061, 278, 915, 461, 120
10	2, 750, 892, 211, 809, 148, 994, 633, 229, 926, 400
11	19, 464, 657, 391, 668, 924, 966, 616, 671, 344, 752, 852, 992, 000

The exact cardinality of possible minimal support equilibrium strategies is unknown for tournaments bigger than $n=11$. However, a lower bound on this set can be obtained from Shao and Wei (1992), which establish the cardinality of the set of possible Latin squares *C* to be at least $C>\prod _{k}^{n}(k!)^{\frac{n}{k}}$. The cardinality of the set of half-normalized Latin squares equals (1/*n*!)*C* as shown in, e.g., Colbourn and Dinitz (1996). Hence, the set of possible minimal support equilibrium strategies is at least

$$\begin{aligned} \frac{1}{n!}\prod _{k}^{n}(k!)^{\frac{n}{k}}, \end{aligned}$$

which is strictly increasing in *n*. Observe that for tournaments of size $n>2$, each team already has two possible minimal support equilibrium strategies. As shown below, the existence of two or more such strategies guarantees a variety of possible mixed strategy equilibria in this game.

#### Proposition 5

A strategy profile $\beta$ which is a convex combination over the set of minimal support equilibrium strategies, defined in Proposition 4, is an optimal strategy of the game.

Before moving on to the next section, we briefly summarize some of our results. Proposition 4 and Proposition 5 rely entirely on the payoff matrix characteristics—namely (PSE)—and the combinatorial structure of the strategy spaces. The results established by these propositions thus apply also to different forms of payoff representation. As illustrated in Proposition 5, tournaments of size $n>2$ feature an infinite set of equilibrium strategies. This result is in sharp contrast to Hamilton and Romano (1998), who argue that the occurrence of multiple equilibria is a rare case. The results obtained from Propositions 2 and 3 pin down common properties of equilibrium strategies in games satisfying (LSP). Restricting our attention to such games, we may use the observation that in equilibrium, every competitor is assigned with the same probability to every playing slot to address questions of optimal team training in the following section.

## Optimal team training

In this section, we analyze the problem of optimally investing training capacities in the framework of the introduced Sumo coach problems. We frame this analysis as a problem of optimal allocation of scarce resources since we consider it costly to alter a team’s skill. Based on the previous section, we use common properties of equilibrium strategies (in particular Proposition 3) to facilitate the analysis of the most effective alterations in the underlying team’s skill distributions, presupposing equilibrium play of both teams. We assume that such modifications come at some symmetric cost and render this analysis as a bounded maximization problem.

We restrict our analysis to games where the payoff matrix satisfies (LSP) to avoid equilibrium behavior violating Proposition 3. Recall that in such games, all equilibria share the common characteristic that every competitor is assigned in expectation with the same probability to every playing slot. Assuming equilibrium play, thus, every competitor is confronted in their match with the same likelihood to every opposing competitor. Hence, a competitor wins (when competitors’ strengths are realized) with probability

$$\begin{aligned} \bar{q}(\theta ^A)=\frac{1}{n}\sum _{j=1}^{n}q(\theta ^A,\theta _j^B). \end{aligned}$$

Fixing equilibrium play of team *B* and given two team’s skill densities $f^A$ and $f^B$, the expected probability that competitor *i* wins their match is

$$\begin{aligned} \mu _{i}(\Theta ^A_{(i)},f^B)= \frac{1}{n}\sum _{j=1}^{n}p(\Theta _{(i)}^A,\Theta _{(j)}^B), \end{aligned}$$

by linearity of the expectation. We will sometimes refer to (expected) marginal increases in a competitor’s winning probability as a function of individual competitor strength. In a slight abuse of notation, we define

$$\begin{aligned} q^{\prime } (\theta ^A,\theta ^B)=\frac{\partial q(\theta ^A,\theta ^B)}{\partial \theta ^A}. \end{aligned}$$

The expected increase in a competitors winning probability is thus

$$\begin{aligned} \mu _{i}^{\prime }(\Theta ^A_{(i)},f^B)=\frac{1}{n}\sum _{j=1}^{n}\mathbb {E}[q^{\prime } (\Theta _{(i)},\Theta _{(j)}]. \end{aligned}$$

Assuming equilibrium play of team *A*, it follows furthermore from Proposition 3 that the expected probability that team *A* wins the tournament can be written as

7 $$\begin{aligned} \mu (f^A,f^B)= \frac{1}{n}\sum _{i=1}^{n}\frac{1}{n}\sum _{j=1}^{n} p(\Theta _{(i)}^A,\Theta _{(j)}^B). \end{aligned}$$

We formalize the concept of “training” as a bounded modification of team *A*’s skill density function $f^A$, i.e., by transferring probability mass among the support interval. We denote the “training capacity” available to a team by $\varepsilon \in \mathbb {R_+}$. Let $f_0^A$ represent the skill density function of the “untrained” team, and let $f_1^A$ denote a team’s skill density function after applying training of amount $\varepsilon$.^7^ Fixing team *B*’s skill distribution, team *A*’s coach maximizes her probability of winning the tournament by solving the following maximization problem

8 $$\begin{aligned} \begin{array}{rlc} \underset{f^A_1 \in \Sigma ^+}{\text {max}}&{}\mu (f^A_1,f^B)\\ \text {s.t.}&{}d(f^A_1,f^A_0) \le \varepsilon \\ \end{array} \end{aligned}$$

in which $\Sigma ^+$ denotes the set of all sigma-additive probability measures with finite support $[0,\bar{\theta }]$, and where $d(\cdot )$ represents an appropriate distance measure between $f^A_0$ and $f^A_1$. Observe that a solution to this maximization problem, $\breve{f}^A_1$, first-order stochastically dominates $f_0^A$, i.e.,

$$\begin{aligned} \breve{F}_1^A(\theta )\le F_0^A(\theta ), \forall \theta {\in }[0,\bar{\theta }]\quad \text {and}\quad \exists \theta {\in }[0,\bar{\theta }] {:}\breve{F}_1^A(\theta ){<}F_0^A(\theta ). \end{aligned}$$

This follows from the observation that it cannot be optimal for a coach to transfer probability mass from “high” strength (and therefore relatively high winning probability) to “low” (with relatively low winning probability). However, what distance measures may be perceived as adequate deserves some further explanation. Conventional distance measures between functions, e.g., the euclidean distance, fail to address our intuition that training somehow has to reflect gradual improvements in a team’s skill distribution. We use the intuition that the cost of transfer of probability mass (i.e., training) between two points has to depend (i) on the amount of probability mass and (ii) on the distance between those points. We thus assume that a gradual increase in competitor strength comes at a lower cost than a radical increase. A measure that naturally commits to these requirements is the Wasserstein distance. Commonly known under the term “earth mover’s distance,” it refers to the minimum cost of transferring probability mass among the support interval when distance and mass of the transfer matter. In the case of one-dimensional probability measures, we follow Vallender (1974) and define $d(\cdot )$ as

$$\begin{aligned} d(f^A_1,f^A_0)=\int _0^{\bar{\theta }}|F^A_1(x)-F^A_0(x)|\mathrm {d}x. \end{aligned}$$

We illustrate the difference between the conventional Euclidean distance and the Wasserstein distance in the following example.

### Example 5

**Fig. 1:**
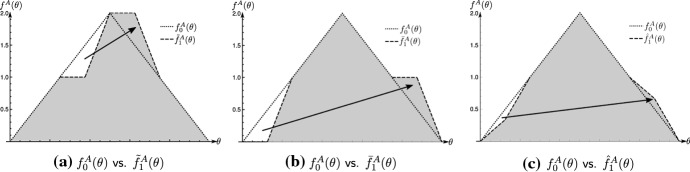
Optimal training of team skill, using the values of Example 1, with parameters $T=7$, $\varepsilon =1/128$, $r=1$

Consider again team *A*’s skill density function $f_0^A(\theta )$ defined to be the symmetric triangular distribution on the unit interval, denoted by $\mathcal {T}[0,1]$. Furthermore we define three trained skill distributions, denoted by $\tilde{f}_1^A(\theta ),\bar{f}_1^A(\theta )$ and $\hat{f}_1^A(\theta )$. Figure 1a illustrates a gradual increase of middle competitor strengths; in Fig. 1b, the same mass is shifted from low to high regions of competitor strength, and in Fig. 1c only a fraction of the mass is shifted from low to high regions of competitor strength. By intuition, $\bar{f}_1^A(\theta )$ yields the highest gain in winning probabilities for team *A*. Now denote by $d_1(\cdot )$, the usual Euclidean distance, and by $d_2(\cdot )$ the Wasserstein distance, as defined above. Embedding the Euclidean distance gives training capacities $d_1(f_0^A,\tilde{f}_1^A)=d_1(f_0^A,\bar{f}_1^A)>d_1(f_0^A,\hat{f}_1^A)$. The distance of the transfer of probability mass is not reflected using the Euclidean, since training that only gradually increases competitor strength requires the same amount of training capacity as training that drastically increases the same mass. In contrast, embedding the Wasserstein distance gives capacities $d_1(f_0^A,\bar{f}_1^A)>d_1(f_0^A,\tilde{f}_1^A)=d_1(f_0^A,\hat{f}_1^A)$. To radically increase competitor strength, such training has to compensate with the transfer of less probability mass.

To obtain finite degrees of freedom, we reformulate the maximization problem by representing $f^A_1$ with some finitely discretized function $\tilde{f}_1^A$. We do so by splitting the probability density into $T\in \mathbb {N}$ kernel densities

$$\begin{aligned} \tilde{f}_1^A=\frac{1}{T*h}\sum _{k=1}^{T}a_k \phi \left( \frac{x-x_k}{h}\right) \hbox { with }a_k\ge 0,\hbox { and } \sum _{k=1}^{T}a_k=T+1. \end{aligned}$$

We define $\phi (\cdot )$ to be an appropriate kernel density function, *h* an appropriate smoothing bandwidth parameter and $x_k=k \bar{\theta }/(T+1)$, $k\in \{1,\ldots T\}$, defined to be the sampling points, distributed equally among the support. The vector $a=\langle a_1,\ldots ,a_T \rangle$, represents linear weights attached to the *k* kernel densities. Modifying (8) accordingly we obtain

$$\begin{aligned} \begin{array}{rrlc}\displaystyle \underset{a}{\text {max}}&{}\ \mu (\tilde{f}^A_1,f^B)=&{}\frac{1}{n^2}\sum _{i=1}^{n}\sum _{j=1}^{n} p(\tilde{\Theta }_{(i)}^A,\Theta _{(j)}^B)&{}\\ \text {s.t.}&{}c_1: &{}d(\tilde{f}_1^A,f_0^A)\le \varepsilon \\ &{}c_2: &{}a_k\ge 0, &{}\forall k \in \{1,\ldots ,T\} \\ &{}c_3: &{}\sum _{k=1}^{T}a_k=T+1, \end{array} \end{aligned}$$

where $\tilde{\Theta }^A_{(i)}$ is defined as the $i^{th}$ order statistic derived from the discretized density $\tilde{f}_1^A$. The constraint $c_1$ bounds possible modifications of $f_0^A$ by $\varepsilon$, embedding the Wasserstein metric. The constraint $c_2$ ensures positive density of $\tilde{f}_1^A$, while $c_3$ ensures $\int _{0}^{\bar{\theta }}\tilde{f}^A_1(x)dx=1$.

### Example 6

We illustrate the solution to this maximization problem, using the teams’ skill distributions of Example 1 and embed the lottery CSF with an discriminatory extent of $r=1$ as the probability to win a distinct match. Fixing team *B*’s skill to $\mathcal {U}[0,1]$, team *A*’s probability to win the tournament, given its original skill distribution $\mathcal {T}[0,1]$, is

$$\begin{aligned} \mu (\mathcal {T}(0,1),\mathcal {U}[0,1])= \frac{1}{n^2}\sum _{i=1}^{n}\sum _{j=1}^{n}\mathbb {E}\left[ \frac{(\Theta _{(i)}^A)^r}{(\Theta _{(i)}^A)^r+(\Theta _{(j)}^B)^r}\right] \approx 0.527. \end{aligned}$$

We use $T=7$ discretization points $x_k$ in the unit interval [0, 1], by setting $x_k=k/8,k=1,\ldots ,7$ and define triangular kernel densities, by setting

$$\begin{aligned} \phi (y)= {\left\{ \begin{array}{ll} (1-|y|) &{} |y|\le 1 \\ 0 &{} \text {otherwise} \\ \end{array}\right. } \end{aligned}$$

and set the corresponding bandwidth to $h=1/(T+1)$.^8^ Observe that we can represent $f^A_0$ precisely with a discretized function $\tilde{f}_0^A$, using the definitions above and setting the weighting vector to

$$\begin{aligned} 2a=\left\langle 1,2,3,4,3,2,1\right\rangle . \end{aligned}$$

We approach this optimization problem numerically since the integral in the constraint $c_3$

$$\begin{aligned} d(f_1^A,f_0^A)=\int _0^{\bar{\theta }}|\tilde{F}^A_1(x)-F^A_0(x)|\mathrm {d}x, \end{aligned}$$

with

$$\begin{aligned} \tilde{F}^A_1(x)=\frac{1}{T*h}\sum _{k=1}^{T}a_k \int _{0}^{x}\phi \left( \frac{x-x_k}{h}\right) \mathrm {d}x, \quad F^A_0(x)= {\left\{ \begin{array}{ll} 4 x &{} 0 \le x \le 0.5 \\ 4 (1-x) &{} 0.5 < x \le 1\\ \end{array}\right. }, \end{aligned}$$

has, to the best of our knowledge, no analytical solution. We integrate numerically using Monte Carlo integration and search for a global maximum using the method of “Differential Evolution,” a stochastic function minimizer.^9^ Solutions to the maximization problem heavily depend on the initial skill densities $f^A$, $f^B$, and the exogenous value of *r*, which parametrizes the functional behavior of $\mu (f^A,f^B)$. Setting $\varepsilon =1/128$, maximization with respect to $a_i$ gives

$$\begin{aligned} 2a=\left\langle 0,3,3,4,3,2,1\right\rangle , \end{aligned}$$

which shifts the expected competitor strengths from

$$\begin{aligned} \langle \mathbb {E}[\Theta ^A_{(1)}], \mathbb {E}[\Theta ^A_{(2)}], \mathbb {E}[\Theta ^A_{(3)}]\rangle =\langle 0.325, 0.5, 0.675\rangle \end{aligned}$$

to approximately

$$\begin{aligned} \langle \mathbb {E}[\tilde{\Theta }^A_{(1)}], \mathbb {E}[\tilde{\Theta }^A_{(2)}], \mathbb {E}[\tilde{\Theta }^A_{(3)}]\rangle \approx \langle 0.346, 0.503, 0.675\rangle . \end{aligned}$$

The probability, that team *A* now wins the tournament is

$$\begin{aligned} \mu ^A(\tilde{f}_1^A,\mathcal {U}[0,1])\approx 0.535. \end{aligned}$$

**Fig. 2:**
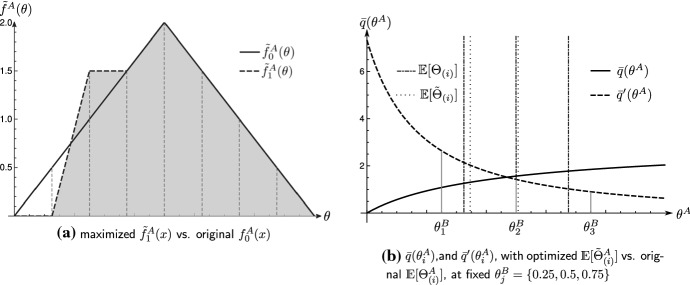
Optimal training of team skill, using the values of Example 1, with parameters $T=7$, $\varepsilon =1/128$, $r=1$

The solution to the optimization problem is illustrated in Fig. 2. As illustrated in Fig. 2a, optimal training transfers probability mass exclusively from the smallest sample point $x_1$ to the second-lowest $x_2$. Optimal training, hence, prioritizes areas of low competitor strength. The reason behind this is illustrated in Fig. 2b. Since $q'(\theta ^A,\theta ^B)$ is strictly decreasing in $\theta ^A$, $\mu _i^{ \prime }(\Theta _{(i)}^A,f^B)$ is decreasing monotonically in *i*

$$\begin{aligned} \mu _1^{ \prime }(\Theta _{(1)}^A,f^B)\approx 0.772{> }\mu _2^{ \prime }(\Theta _{(2)}^A,f^B)\approx 0.469{ > }\mu _3^{ \prime }(\Theta _{(3)}^A,f^B)\approx 0.329. \end{aligned}$$

Training that favors $\Theta _{(i)}^A$ yields a higher impact on the overall winning probability $\mu (f^A,f^B)$, than training that favors, e.g., $\Theta _{(2)}^A$. We conclude this example by highlighting that the solution to this maximization problem can be generalized to the parameter space $r\in (0,1]$.

Example 6 demonstrates the linkage between the expected marginal increase of a competitor’s winning probability and the optimal allocation of training capacities. Training is optimally invested in the weakest regions of a team’s skill density if $q^{\prime }(\theta ^A,\theta ^A)$ is strictly decreasing. This follows simply by the observation that such alterations of probability mass, by definition, have the highest impact on the strength of the weakest player $\Theta _{(1)}^A$. We formalize this observation in a concluding proposition.

### Proposition 6

If the embedded CSF $q(\theta ^A,\theta ^B)$ is strictly increasing in $\theta ^A$ and concave, optimal training should prioritize the lowest areas of $\tilde{f}^A$, by decreasing the lowest possible linear weight denoted by $a_{min}$ and increase the second to lowest weight $a_{min+1}$, iteratively under the constraint $a_k\ge 0$.

In the last example of this section, we illustrate that the concavity of the embedded CSF is indeed necessary for Proposition 6.

### Example 7

We use the same parameters as in Example 6, and alter the discriminatory extent to $r=20$. Doing so, the embedded Tullock CSF is not concave, a property which transfers to the expect change in probability to win the tournament $\mu ^{ \prime A}(\Theta _{(i)}^A,f^B)$. The solution to the maximization problem is

$$\begin{aligned} 2a=\left\langle 1,1,4,4,3,2,1\right\rangle , \end{aligned}$$

which shifts the expected competitor strengths from

$$\begin{aligned} \langle \mathbb {E}[\Theta ^A_{(1)}], \mathbb {E}[\Theta ^A_{(2)}], \mathbb {E}[\Theta ^A_{(3)}]\rangle =\langle 0.325, 0.5, 0.675\rangle \end{aligned}$$

to approximately

$$\begin{aligned} \langle \mathbb {E}[\tilde{\Theta }^A_{(1)}], \mathbb {E}[\tilde{\Theta }^A_{(2)}], \mathbb {E}[\tilde{\Theta }^A_{(3)}]\rangle \approx \langle 0.341, 0.507, 0.676\rangle \end{aligned}$$

The probability that team *A* now wins the tournament shifts from

$$\begin{aligned} \mu (f_1^A,f^B)\approx 0.502 \quad \text {to} \quad \mu (f_1^A,f^B)\approx 0.510 \end{aligned}$$

**Fig. 3:**
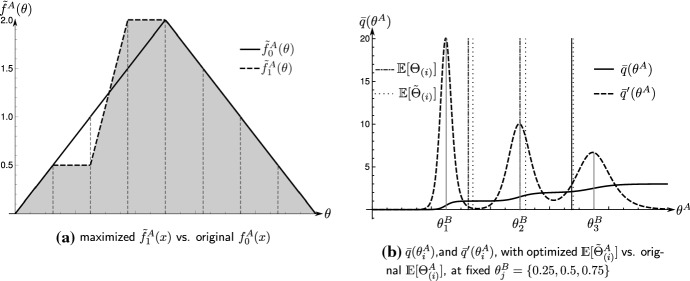
Optimal training of team skill, using the values of Example 1, with parameters $T=7$, $\varepsilon =1/128$, $r=20$

The solution to the optimization problem is illustrated in Fig. 3. As illustrated in Fig. 3a, optimal training now transfers probability mass exclusively onto the central discretization point $x_5$. Optimal training now prioritizes areas of middle competitor strength. The intuition for this is illustrated in Fig. 3b. Since $\mu _2^{\prime }(\Theta ^A_{(2)},f^B)=1.003$ is greater than $\mu _1^{ \prime A}(\Theta ^A_{(1)},f^B)=1.002$ and $\mu _3^{ \prime }(\Theta _{(3)}^A,f^B)=0.980$, respectively, training that favors $\Theta _{(2)}^A$ now yields a higher impact on the overall winning probability $\mu (f^A,f^B)$.

The observation that optimal training should be invested in areas where the marginal increase in a competitor’s winning probability is maximal follows conventional economic arguments based on marginal revenues. In this context, the analysis of the Tullock CSF form also gives room for further interpretations. As illustrated in Example 6, training in tournaments that involve a high degree of luck (i.e., low values of *r*) should prioritize the weakest players. On the other hand, if the tournament is highly decisive regarding the player strengths (i.e., high values of *r*), the team should apply the training in areas where a team’s expected strength realizations are closest to the opposing team’s expected strength realizations. In summary, the analysis in this section addresses the question of optimal team training superficially. Although the introduced mechanics provide exciting insights into this problem, the established result applies to a narrow class of contests. We refrain from a more systematic analysis of optimal team training as this seems to diverge from the primary interest of this paper.

## Concluding remarks

The present paper analyzes, first, the optimal seeding of resource bundles in the setting of a multi-battlefield contest and, second, the optimal transfer of additional resources between such sets of bundles controlled by one player. An immediate result of our equilibrium characterization is that Nash equilibria must result in a “totally” randomized allocation of resource bundles with respect to the distinct single-match contests. Although this characteristic is not surprising in a finite zero-sum game, our result shows that such realizations can be achieved through multiple probability distributions over the pure strategy space. The results of this paper diverge quite substantially from empirical observations of real sports competitions where the overwhelming majority of teams seem to be indexed decreasing in strength. As Hamilton and Romano (1998) show and we confirm in a more general setting, however, such a seeding strategy can only be optimal over trivial subsets of the parameter space. Possible extensions of our model include unequal valuations of the distinct playing slots, either symmetric for both players, or asymmetrically, rendering the zero-sum designation problematic. Dynamic considerations could be addressed by formalizing a “Stackelberg” sequential version of the game, in which one team moves first. This would yield different results in the comparative static analysis with optimal training dependent on the first- or second-mover position, leading to perhaps interesting industrial organization or political economy applications. As our analysis abstracts from the players’ effort choices, another possible extension could negatively link the players’ choice of efforts to the strength differential in a single contest and thereby address motivational issues in team sports.

## Appendix

### Proof of Lemma 1

(P1): this property simply follows by linearity of the Expectation:

$$\begin{aligned}&\mathbb {E}[q^A (\Theta _{(i)}^B,\Theta _{(j)}^B)]+\mathbb {E}[q^B (\Theta _{(i)}^B,\Theta _{(j)}^B)]=1\\&\quad =\mathbb {E}[q^A (\Theta _{(i)}^B,\Theta _{(j)}^B)+q^B (\Theta _{(i)}^B,\Theta _{(j)}^B)]=1 \end{aligned}$$

by (Q1) we have $q^A(\theta ^A,\theta ^B)+q^B(\theta ^A,\theta ^B)=1$, and thus $\mathbb {E}[1]=1$.

(P2): Set $\Theta ^A_{(i)}=\Theta ^B_{(j)}$, we have

$$\begin{aligned} \mathbb {E}[q^A (\Theta _{(i)}^A,\Theta _{(j)}^B)]=\mathbb {E}[q^A (\Theta _{(i)}^A,\Theta _{(i)}^A)] \end{aligned}$$

by (Q2) we have $q^A(\theta ^A,\theta ^B)=1/2$ for identical qualities and thus $\mathbb {E}[1/2]=1/2$.

(P3): W.L.O.G. set $1<k<l<n$, which gives $\Theta _{(k)}^A<_{s.t.}\Theta _{(l)}^A$ (see, e.g., Shaked and Shanthikumar (2007)). Now construct $Y=q^A(\Theta _{(k)}^A,\Theta _{(j)}^B)$ and $Z=q^A(\Theta _{(l)}^A,\Theta _{(j)}^B)$. Since $q^A(\cdot )$ is increasing in its first argument by (Q3), we have $Y<_{s.t.}Z$, giving in turn the inequality on the expectations $\mathbb {E}[Y]<\mathbb {E}[Z]$.

### Proof of Lemma 2

Recall that the probability that team *A* wins the tournament, given a distinct lineup $(a_x,b_y)$, (2), is

$$\begin{aligned} \rho (a_x,b_y) = \frac{1}{n}\sum _{k=1}^{n}p(\Theta (a_x(k)),\Theta (b_y(k))). \end{aligned}$$

Let $\Theta _{(i)}$ indicate the stochastic player strength of team *A*’s player *i*, assigned to slot $\bar{k}$ by strategy $a_x$. Let $\Theta _{(j)}$ indicate the stochastic player strength of team *B*’s player *j*, assigned to slot $\bar{k}$ by strategy $b_y$. Further, let $\Theta _{(i')}$ indicate the stochastic player strength of team *A*’s player $i'$, assigned to slot $\bar{k}'$ by strategy $a_x$ and let $\Theta _{(j')}$ indicate the expected player strength of team *B*’s player $j'$, assigned to slot $\bar{k}'$ by strategy $b_y$. Following (1), the lineup $(a_x,b_y)$ is

$$\begin{aligned} (a_x,b_y)= & {} \langle [\Theta (a_x(1)),\Theta (b_y(1))],\ldots ,[\Theta _{(i)},\Theta _{(j)}],,\ldots ,,[\Theta _{(i')}, \Theta _{(j')}],\ldots ,\\&\quad [\Theta (a_x(n)),\Theta (b_y(n))]\rangle . \end{aligned}$$

The payoff of lineup $(a_x,b_y)$ is

$$\begin{aligned}&\rho (a_x,b_y) \\&\quad = \frac{1}{n}\left( p(\Theta _{(i)},\Theta _{(j)})+p(\Theta _{(i')},\Theta _{(j')})+\sum _{k\in N \setminus \{\bar{k},\bar{k}'\}}p(\Theta (a_x(k)),\Theta (b_y(k)))\right) . \end{aligned}$$

Now construct lineup $(a_{x'},b_{y'})$, such that the players assigned to slot $\bar{k}$ in $(a_{x},b_{y})$ are now assigned to slot $\bar{k}'$ and vice versa. $\Theta _{(i)}$ thus indicates the expected player strength of team *A*’s player *i*, assigned to slot $\bar{k}'$ by strategy $a_{x'}$. $\Theta _{(j)}$ indicates the expected player strength of team *B*’s player *j*, assigned to slot $\bar{k}'$ by strategy $b_{y'}$. Further, $\Theta _{(i')}$ indicates the expected player strength of team *A*’s player $i'$, assigned to slot $\bar{k}$ by strategy $a_{x'}$, while $\Theta _{(j')}$ indicates the expected player strength of team *B*’s player $j'$, assigned to slot $\bar{k}$ by strategy $b_{y'}$. Lineup $(a_{x'},b_{y'})$ is then

$$\begin{aligned} (a_{x'},b_{y'})= & {} \langle [\Theta (a_{x'}(1)),\Theta (b_{y'}(1))],\ldots , [\Theta _{(i')},\Theta _{(j')}],,\ldots ,,[\Theta _{(i)},\Theta _{(j)}],\ldots ,\\&\quad [\Theta (a_{x'}(n)),\Theta (b_{y'}(n))]\rangle . \end{aligned}$$

The payoff of lineup $(a_{x'},b_{y'})$ is

$$\begin{aligned}&\rho (a_{x'},b_{y'}) \\&\quad = \frac{1}{n}\left( p(\Theta _{(i')},\Theta _{(j')})+p(\Theta _{(i)},\Theta _{(j)})+\sum _{k\in N \setminus \{\bar{k},\bar{k}'\}}p(\Theta (a_x(k)),\Theta (b_y(k)))\right) . \end{aligned}$$

Consequently $\rho (a_{x'},b_{y'})$ obviously equals $\rho (a_{x},b_{y})$, and is therefore non-injective.

### Proof of Lemma 3

As illustrated in Lemma 2, lineups that are only permutations over *k* yield the same payoff tuples. The set of non-equivalent lineups with respect to payoffs can be constructed, using (4) and arbitrarily fixing any pure strategy of team *B*

$$\begin{aligned} \mathscr {L}_i=\{(a_x,b_y)|(a_x,b_y)\text { is a permutation of }(a_x,b_i)\text { over }k\}. \end{aligned}$$

An equivalent partition, $\mathscr {L}$, can be constructed by arbitrarily fixing any pure strategy of team *A*

$$\begin{aligned} \mathscr {L}_j=\{(a_x,b_y)|(a_x,b_y)\text { is a permutation of }(a_j,b_y)\text { over }k\}. \end{aligned}$$

Consequently, each row and each column of $\Phi$ contains the same elements, and therefore satisfies the definition of (PSE). The second part of the statement follows simply by the definition of (LSP), an the observation that payoffs in each row and column are unique, according to the strict order relation.

### Proof of Lemma 4

Following Ferguson (2020), the value of the game is exactly the expected payoff when both players use their optimal mixed strategies, i.e.,

$$\begin{aligned} V=\sum _{x=1}^{n!}\sum _{y=1}^{n!}\alpha _x\rho (a_x,b_y)\beta _y. \end{aligned}$$

Plugging in equal probability mixing (i.e., $\alpha _x=1/n!=\beta _y$) over the entire pure strategy spaces, which always constitutes an equilibrium of games satisfying (PSE), yields

9 $$\begin{aligned} V=\frac{1}{n!}\sum _{x=1}^{n!}\frac{1}{n!}\sum _{y=1}^{n!}\rho (a_x,b_y). \end{aligned}$$

By (PSE) we have

10 $$\begin{aligned} \frac{1}{n!}\sum _{x=1}^{n!}\rho (a_x,b_y)=\frac{1}{n!}\sum _{y=1}^{n!}\rho (a_x,b_y), \end{aligned}$$

for arbitrary choices of *x* or *y*. Plugging (10) into ((9)) results in

11 $$\begin{aligned} V=\frac{1}{n!}\sum _{x=1}^{n!}\frac{1}{n!}\sum _{x=1}^{n!}\rho (a_x,b_y)=\frac{1}{n!}\sum _{x=1}^{n!}\rho (a_x,b_y), \end{aligned}$$

which concludes the proof.

### Proof of Proposition 1

We develop the proof in two steps. We establish sufficiency by contraposition, assuming that (5) does not hold and show that such a strategy profile cannot be optimal. W.L.O.G. assume that there exists distinct $a_x$ and $a_{x'}$ for which it holds that

12 $$\begin{aligned} \pi (a_x,\beta ^{*})>\pi (a_{x'},\beta ^{*}). \end{aligned}$$

To prove sufficiency, we need to establish that such a strategy $\beta ^{*}$ violates equilibrium properties for the distinct pair $a_x$ and $a_{x'}$. By (PSE), we know that any pure strategy of team *A* leads to every possible payoff pair. Hence, it is always possible to find such a pure strategy pair $a_x$ and $a_{x'}$, if (5) does not hold $\forall a_x\ne a_{x'} \in S^A$. Thus, player *A* always has a response strategy $a_x$ to obtain its preferred payoffs, which coincidentally results in lower payoffs for player *B*. Such a mixed strategy $\beta$ can consequently not be optimal for player *B* in the first hand.

In a second step, we establish necessity, i.e., any strategy profile satisfying (5) is an optimal strategy for player *B*. Following Ferguson (2020), a strategy profile is optimal for Player *B* if and only if Player *A*’s average payoff is at most *V*, no matter what pure strategy player *A* uses, i.e.,

13 $$\begin{aligned} \sum _{y=1}^{n!}\beta _y\rho (a_x,b_y)\le V=\frac{1}{n!}\sum _{x=1}^{n!}\rho (a_x,b_y) \forall y=1,\ldots n!. \end{aligned}$$

Observe that by indifference we have

$$\begin{aligned} \sum _{y=1}^{n!}\beta _y\rho (a_x,b_y)=\sum _{y=1}^{n!}\beta _y \rho (a_{x'},b_y) \forall a_x\ne a_{x'} \in S^A, \end{aligned}$$

and thus also

$$\begin{aligned} \sum _{y=1}^{n!}\beta _y\rho (a_x,b_y)=\sum _{y=1}^{n!}\beta _y \frac{1}{n!}\sum _{x=1}^{n!}\rho (a_x,b_y)=\sum _{y=1}^{n!}\beta _yV=V, \end{aligned}$$

establishing the equality in (13). The proof for Player *B* follows by symmetry.

### Proof of Proposition 2

In a first step we establish sufficiency. Let $a_x$ denote some strategy that assigns a competitor with stochastic strength $\Theta _{(i)}^A$ to slot *k* and the competitor with stochastic strength $\Theta _{(j)}^A$ to slot $k'$. Let $a_{x'}$ denote the strategy that assigns the competitor with stochastic strength $\Theta _{(i)}$ to playing slot $k'$ and the competitor with stochastic strength $\Theta _{(j)}$ to playing slot *k*, while all other assignments are kept equal. Following Proposition 2, we set:

$$\begin{aligned} \pi (a_{x},\beta ,k)= & {} \frac{1}{n}\sum _{y=1}^{n!}\beta _y p(\Theta _{(i)}^A,\Theta (b_y(k)))\\= & {} \frac{1}{n}\sum _{y=1}^{n!}\beta _y p(\Theta _{(i)}^A,\Theta (b_y(k')))=\pi (a_{x'},\beta ,k'), \\ \pi (a_{x},\beta ,k')= & {} \frac{1}{n}\sum _{y=1}^{n!}\beta _y p(\Theta _{(j)},\Theta (b_y(k)))\\= & {} \frac{1}{n}\sum _{y=1}^{n!}\beta _y p(\Theta _{(j)},\Theta (b_y(k')))=\pi (a_{x'},\beta ,k). \end{aligned}$$

Observe that summing (6) over all playing slots exactly yields the expected payoff $\pi (a_x,\beta )$, hence we have

$$\begin{aligned} \pi (a_x,\beta )= & {} \frac{1}{n}\sum _{y=1}^{n!}\left( \beta _y \sum _{k=1}^{n} p(\Theta (a_x(k)),\Theta (b_y(k)))\right) =\frac{1}{n}\sum _{k=1}^{n}\pi (a_{x},\beta ,k)\\= & {} \frac{1}{n}\sum _{k=1}^{n}\pi (a_{x'},\beta ,k)\\= & {} \frac{1}{n}\sum _{y=1}^{n!}\left( \beta _y \sum _{k=1}^{n} p(\Theta (a_{x'}(k)),\Theta (b_y(k)))\right) =\pi (a_{x'},\beta ), \end{aligned}$$

repeating for some other slot $k'$ or some other starting strategy $a_x$ establishes Proposition 1.

Necessity is shown by contradiction. W.L.O.G. assume that for two playing slots *i* and *j* it holds that

$$\begin{aligned} \sum _{y=1}^{n!}\beta _y p(\Theta _{(i)}^A,\Theta (b_y(i)))>\sum _{y=1}^{n!}\beta _y p(\Theta _{(i)}^A,\Theta (b_y(j))). \end{aligned}$$

Since $\Theta _{(i)}^A$ achieves a higher payoff on slot *i* than on slot *j*, the difference in payoff must be a consequence of the mixed strategy $\beta$. Recall that $p(\Theta _{(i)}^A,\Theta (b_y(j)))=\mathbb {E}[q(\Theta _{(i)}^A,\Theta (b_y(j)))]$ is strictly decreasing in *j* by Lemma 1. Hence we have

$$\begin{aligned} \sum _{y=1}^{n!}\beta _y\Theta (b_y(i)))<_{\text {s.t.}}\sum _{y=1}^{n!}\beta _y\Theta (b_y(j))). \end{aligned}$$

In such a strategy profile $\beta$, some unambiguously stronger player (denoted by $\Theta _{(i)}^B$) must be seeded with a higher probability to slot *j* than to slot *i*. Likewise, some unambiguously weaker player (denoted by $\Theta _{(j)}^B$) must be seeded with a higher probability to slot *i* than to slot *j*. Now arbitrary pick some strategy $b_y$ such that $\Theta (b_y(j))=\Theta _{(i)}^B$ and $\Theta (b_y(i))=\Theta _{(j)}^B$ and pick two pure strategies of player *A*, denoted by $a_i$ and $a_j$, that assign $\Theta (a_i(i))=\Theta (a_j(j))=\Theta _{(i)}^A$ and $\Theta (a_i(j))=\Theta (a_j(i))=\Theta _{(j)}^A$, and keep every assignment on the other slots unchanged. By (LSP), we have either $\rho (a_i,b_y)>\rho (a_j,b_y)$ or $\rho (a_i,b_y)<\rho (a_j,b_y)$ since $(a_i,b_y)$ and $(a_j,b_y)$ are elements of distinct lineup classes. Hence, for the mixed strategy $\beta$, we must thus also have either $\pi (a_i,\beta )>\pi (a_j,\beta )$ or $\pi (a_i,\beta )<\pi (a_j,\beta )$. Such a mixed strategy profile by definition contradicts Proposition 1.

### Proof of Proposition 3

In a first step, we establish sufficiency. Recall that a sufficient condition for a mixed strategy profile $\beta$ being optimal is

$$\begin{aligned} \pi (\Theta _{(i)}^A,\beta ,k)= & {} \frac{1}{n}\sum _{y=1}^{n}\beta _yp(\Theta _{(i)}^A,\Theta (b_y(k)))\\= & {} \frac{1}{n}\sum _{y=1}^{n}\beta _yp(\Theta _{(i)}^A,\Theta (b_y(k')))=\pi (\Theta _{(i)}^A,\beta ,k'). \end{aligned}$$

Let $S_{j:k}^B$ denote the set of $(n-1)!$ strategies, which assign some competitor $\Theta _{(j)}^B$ to playing slot *k*. The probability that competitor $\Theta _{(j)}^B$ is assigned to slot *k*, given some mixed strategy vector $\beta$, is

14 $$\begin{aligned} \sum _{y\in S_{j:k}}^{(n-1)!}\beta _y=\beta _{j:k} \hbox { with } \displaystyle {\sum _{k=1}^{n}\beta _{j:k}=1=\sum _{j=1}^{n}\beta _{j:k}}. \end{aligned}$$

Observe that the payoff on slots *k* and $k'$ is

$$\begin{aligned}&\pi (\Theta _{(i)}^A,\beta ,k)=\frac{1}{n}\sum _{y=1}^{n}\beta _yp(\Theta _{(i)}^A,\Theta (b_y(k)))=\sum _{j=1}^{n}\beta _{j:k}p(\Theta _{(i)}^A,\Theta _{(j)}^B) \hbox { and } \\&\pi (\Theta _{(i)}^A,\beta ,k')=\frac{1}{n}\sum _{y=1}^{n}\beta _yp(\Theta _{(i)}^A,\Theta (b_y(k')))=\sum _{j=1}^{n}\beta _{j:{k'}}p(\Theta _{(i)}^A,\Theta _{(j)}^B). \end{aligned}$$

Setting $\beta _{j:k}=\beta _{j:k'}\forall j\in N$ establishes the equality in (14). Necessity follows from the same argument as in the proof of Proposition 2.

### Proof of Proposition 4

Suppose a mixed strategy $\beta$ which consists out of two parts. Let $\hat{b}_y$ denote a pure strategy of team *B* of some proper subset $\hat{S}^{B}$, as defined in Proposition 4. Let $\tilde{b}_y\in S^B\setminus \hat{S}^B$, and let $\tilde{\beta }_y$ denote a mixed strategy dimension of $\beta$ associated with a pure strategy $\tilde{b}_y$. Team *A*’s payoff obtained on a distinct playing slot *k*, given its strategy $a_x$ and such a mixed strategy $\beta$, can be written as

15 $$\begin{aligned}&\pi (a_x,\beta ,k) \nonumber \\&\quad =\frac{1}{n}\left( \sum _{y\in \hat{S}}\hat{\beta }_y p(\Theta (a_x(k)),\Theta (\hat{b}_y(k)))+\sum _{y\in S\setminus \hat{S}}\tilde{\beta }_y p(\Theta (a_x(k)),\Theta (\tilde{b}_y(k)))\right) .\nonumber \\ \end{aligned}$$

Setting $\hat{\beta }_y=1/n$ and $\tilde{\beta }_y=0$ according to Proposition 4, simplifies (15) to

$$\begin{aligned} \pi (a_x,\hat{\beta },k) =\frac{1}{n^2}\sum _{y\in \hat{S}} (\Theta (a_x(k)),\Theta (\hat{b}_y(k))). \end{aligned}$$

Observe that

$$\begin{aligned} \pi (\Theta _(i),\hat{\beta },k)&=\frac{1}{n^2}\sum _{y\in \hat{S}} p(\Theta _{(i)},\Theta (\hat{b}_y(k)))=\frac{1}{n^2}\sum _{y\in \hat{S}} p(\Theta _{(i)},\Theta (\hat{b}_y(k')))\\&=\pi (\Theta _{(i)},\hat{\beta },k') \end{aligned}$$

holds, since by definition of Proposition 4, the functions

$$\begin{aligned} \{\Theta (\hat{b}_1(k)),\ldots ,\Theta (\hat{b}_{n}(k))\} \text { and } \{\Theta (\hat{b}_1(k')),\ldots ,\Theta (\hat{b}_{n}(k'))\} \end{aligned}$$

evaluate both in permutations of the same *n*-set $\{\Theta _{(1)}^B,\ldots ,\Theta _{(n)}^B\}$ of competitors. $\square$

### Proof of Proposition 5

We prove Proposition 5 by showing that a convex combination of two minimal support equilibrium strategies satisfies Proposition 2, convex combinations of 3 or more minimal support equilibrium strategies follow analogously, but otherwise just complicate the notation. Let $\hat{b}_y\in \hat{S}^{B}$, and $\tilde{b}_y\in \tilde{S}^{B}$ denote pure strategies of team *B* of two (not necessarily disjoint) proper subsets $\hat{S}^{J}$ and $\tilde{S}^{J}$ that satisfy Proposition 4. Let $\hat{\beta }_y$ and $\tilde{\beta }_y$ denote the mixed strategy dimensions of $\beta$ associated to pure strategy $\hat{b}_y$ and $\tilde{b}_y$. According to Proposition 4 we set $\hat{\beta }_y=1/n=\tilde{\beta }_y$. Team *A*’s payoff obtained on a distinct playing slot *k*, given its strategy $a_x$ and team *B*’s strategy $\beta$ is then:

$$\begin{aligned}&\pi (\Theta _{(i)},\beta ,k)\\&\quad =\frac{1}{n}\left( \lambda \frac{1}{n}\sum _{y\in \hat{S}}\hat{\beta }_y p(\Theta (a_x(k)),\Theta (\hat{b}_y(k))) \right. \\&\quad \quad \left. +(1-\lambda )\frac{1}{n}\sum _{y\in \tilde{S}}p(\Theta (a_x(k)),\Theta (\tilde{b}_y(k)))\right) \lambda {\in }[0,1]. \end{aligned}$$

Proposition 2 demands

$$\begin{aligned} \pi (\Theta _{(i)},\beta ,k) =\pi (\Theta _{(i)},\beta ,k') \end{aligned}$$

which is indeed satisfied by convexity and since by definition of $\hat{S}^B$ and $\tilde{S}^B$,

$$\begin{aligned}&\{\Theta (\hat{b}_1(k)),\ldots ,\Theta (\hat{b}_{n}(k))\},\{\Theta (\tilde{b}_1(k)),\ldots ,\Theta (\tilde{b}_{n}(k))\}, \text { and } \\&\{\Theta (\hat{b}_1(k')),\ldots ,\Theta (\hat{b}_{n}(k'))\},\{\Theta (\tilde{b}_1(k')),\ldots ,\Theta (\tilde{b}_{n}(k'))\} \end{aligned}$$

evaluate all in permutations of the *n*-set $\{\Theta _{(1)}^B,\ldots ,\Theta _{(n)}^B\}$. $\square$

### Proof of Proposition 6

Let $\delta (\varepsilon )$ represent an infinitesimal possible shift of probability mass from $a_k$ to $a_{k+1}$ under the constraint of $c_3$. Let $a_{min}$ represent $a_{\text {min}\{k\in \{1,\ldots ,T\}:a_k>0\}}$. Since the random strength of the weakest player $\Theta _{(i)}^A$ is the distribution of the minimum, a possible shift of probability mass, represented by $a_{min}-\delta (\varepsilon )$ to $a_{min+1}+\delta (\varepsilon )$, by construction affects mostly the first order statistic (see, e.g., Shaked and Shanthikumar 2007). It is thus, enough to show that $\mu _i^{ \prime }(\Theta _{(i)}^A,f^B)$ is decreasing monotonically in *i*. Since $\mu ^{ \prime A}(\Theta _{(i)}^A,f^B)$ is defined to be the sum

$$\begin{aligned} \frac{1}{n}\sum _{j=1}^{n}\mathbb {E}[q^{\prime } (\Theta _{(i)},\Theta _{(j)}]. \end{aligned}$$

Since $q'(\theta ^A,\theta ^B)$ is strictly decreasing in $\theta ^A$, by concavity, so does $\mathbb {E}[q'(\Theta _{(i)}^A,\Theta _{(j)}^B)]$, by the same argument as in Lemma 1. $\square$

## Footnotes

1 According to Colbourn and Dinitz (1996), *“a latin square of side **n* *is an* $n\times n$ *array in which each cell contains a single element from an* *n**-set **S*, *such that each element occurs exactly once in each row and exactly once in each column.”*

2 An extension to unequal team sizes is uninteresting since unequal team sizes only affect the strength expectations of the distinct competitors. Within the formulation of our model and in the absence of any secondary participation effects, a rational coach with more competitors, however, always enters the tournament with the strongest among them.

3 Note that the strict stochastic order $<_{\text {s.t.}}$ is induced by $f^J>0$ among the support.

4 Following Konrad (2009), contests are defined as competitive situations in which players compete by exerting non-refundable efforts. For an overview of the contest literature, see, for instance, the comprehensive surveys by Konrad (2009), Vojnović (2016), Chowdhury et al. (2019), and Fu and Wu (2019).

5 A formal proof of the first implication is given in Lemma 1.

6 The theory of Latin squares is a research topic in combinatorics, occasionally applied to experimental design. For an overview of the relevant literature see, e.g., Colbourn and Dinitz (1996) and Keedwell and Dénes (2015).

7 Heterogeneity in costs can be addressed by applying some function $c^J(\varepsilon )$.

8 We use $T=7$ discretization points to illustrate the logic of the optimization problem. An increase of discretization points is computationally expensive, but otherwise analytically uninteresting

9 We use this algorithm, due to its relative robustness in problems that have multiple local minima. We set the parameters due to the rules of thumb introduced by Storn (1996). For more technical details. see, e.g., Price et al. (2005).
